# Ohio Medical Convention

**Published:** 1847-06

**Authors:** Dan. Drake

**Affiliations:** Cincinnati


CINCINNATI, May 22, 1847.

*To my Colleagues:*

GENTLEMEN—I returned yesterday from the annual meeting of the *Medical Convention of Ohio*, at Columbus, and propose to give you a short account of its proceedings, which I hope may reach you in time for our next number.

The first meeting of the Convention was held in the month of January, 1835, the next in the same month, 1838; since which they have been held annually for several years in different large towns, but for the last two or three in Columbus, where by common consent they are likely to be continued. Physicians from thirty counties of the State were present, to the number of one hundred and twenty, which is a gain on any previous convention of thirty or forty. I regret that the physicians of Kentucky have not been as successful in this mode of cultivating the profession as those of Ohio. The Convention of 1841 was not followed by any other in the former, but since that time six have been held in the latter State. Of the great utility of such conventions to those who compose them, if to no others, there cannot be a doubt; and when the time may come that they are numerously attended by the physicians of every State in the Union, the profession of the United States will be found advancing in aspiration and intelligence much beyond anything we have as yet seen.

At the late Convention, papers were read by Dr. Dawson on puerperal erysipelas, by Dr. Bœrsler on the bronchitis and pneumonia of children, and on certain injuries of the brain, by Dr. Howard on several surgical operations, and by Dr. Carney on milksickness; which papers were discussed at considerable length. But the professional exercises of the Convention were not limited to these heads. The appropriate treatment of autumnal fever, the convulsions of children, the inhalation of lethean gas before surgical operations, and trephining in epilepsy from injuries of the cranium, were introduced as topics of debate, and were spoken to with earnestness by a number of members. Finally, several gentlemen verbally communicated facts on different subjects, among which were gutta serena and the Cæsarean operation.

A committee was appointed to report to the next Convention on the lethean gas in surgical operations, and another on the propriety of blood-letting in the hæmoptysis attendant on phthisis

Of course a number of motions were made and some papers read on the subject of societies, education, institutions, and the like. Dr. R. Thompson's elaborate paper on a new method of organizing medical schools, both in their faculties and boards of trustees, and Dr. Bœrsler's paper on kindred topics, together with several resolutions, were referred to a committee to report at the next session of the Convention. A resolution of Dr. Schenck for changing the name of county poor-houses to county *infirmaries* was referred to a committee with instructions to memorialize the Legislature. A series of resolutions transmitted to the Convention by Dr. Kreider, in favor of a reenactment of the law to regulate the practice of physic, were referred to a committee with instructions to petition the General Assembly to that end. Dr. R. Thompson offered a resolution for a report on the establishment of orphan asylums, which was also referred. Dr. Landon offered a resolution, also referred to a committee, to report on the best means of managing lunatics in private practice, and on the establishment of a private lunatic asylum. A memorial from Dr. W. Judkins, requesting the Convention to petition the Legislature to pass a law requiring all venders of patent medicines to attach to them a statement of their composition, was referred to a committee. A resolution offered by Dr. Ball, on the subject of the State poll tax on physicians, was likewise referred. Dr. Butterfield, one of the delegates from the Convention to the late National Convention at Philadelphia, made a verbal report of its proceedings; after which, on motion of Dr. Tate, a resolution was adopted requesting the faculties of the medical schools of Ohio to inform the next State Convention how far they have adopted the recommendations of the National Convention.

Invitations as usual were received from the officers of the Ohio penitentiary, lunatic asylum, and schools for the blind and deaf mute, to visit those institutions; but the time (three days) allotted for the session of the Convention was so entirely absorbed by its business, that the invitations could not be complied with. From all that I could learn, the whole of them are in a flourishing condition—that is, are not only well conducted, but, unfortunately, abound in inmates.

The proceedings and papers of the Convention will be published in a few weeks, when I hope you will give such an account of the latter as may be proper. . . . . . In conclusion, I might mention that on the second night of the convention, the physicians of Columbus gave to its members quite a sumptuous entertainment—a hospitality which has grown into a fixed habit with them. Thus the fifth pair of nerves were exercised not less than the seventh, and in the discussion the latter did not send forth a single angry or resentful expression. There was emulation without rivalry, and wit without wine. DAN. DRAKE.

